# Fetal magnetic resonance imaging in the diagnosis of spinal cord neural tube defects: A prospective study

**DOI:** 10.3389/fneur.2022.944666

**Published:** 2022-08-08

**Authors:** Gan Gao, Benzhang Tao, Yanyan Chen, Jiaqi Yang, Mengchun Sun, Hui Wang, Fangbin Hao, Simeng Liu, Minjie Wang, Aijia Shang

**Affiliations:** ^1^Department of Neurosurgery, Chinese People's Liberation Army (PLA) General Hospital, Beijing, China; ^2^Chinese People's Liberation Army (PLA) Medical School, Beijing, China; ^3^Tianjin Medical University, Tianjin, China; ^4^Department of Anesthesiology, The 960th Hospital of the Chinese People's Liberation Army (PLA), Taian, China

**Keywords:** spinal neural tube defects, fetal, ultrasound, MRI, prenatal diagnosis

## Abstract

**Objective:**

This study aimed to evaluate the value of fetal magnetic resonance imaging (MRI) in the prenatal diagnosis of spinal neural tube defects.

**Methods:**

From August 2018 to January 2021, 56 fetuses with suspected spinal cord neural tube defects were treated by prenatal ultrasound in the Neurosurgery Department of the First Medical Center of the People's Liberation Army General Hospital. Fetal MRI was performed within 72 h after ultrasound diagnosis. Forty singleton fetuses were selected. Magnetic resonance examination was performed within 1 month after birth, and the diagnostic coincidence rates of prenatal ultrasound and fetal magnetic resonance examination in the prenatal diagnosis of spinal cord neural tube defects were compared and analyzed using postnatal magnetic resonance examination as the standard.

**Results:**

The coincidence rates of prenatal ultrasound and fetal MRI for the prenatal diagnosis of spina bifida were 71.4% (20/28) and 39.2% (11/28), respectively, and the difference was statistically significant. The coincidence rates of prenatal ultrasound and fetal MRI in the diagnosis of intraspinal lipoma were 52.6% (10/19) and 73.7% (14/19), respectively, and the difference was statistically significant.

**Conclusion:**

Fetal MRI has an advantage over prenatal ultrasound in detecting intraspinal lipoma. Prenatal ultrasound has an advantage over fetal MRI in detecting spina bifida.

## Introduction

Spinal neural tube defects refer to the lesions occurring in the spinal neural tube, including spina bifida, simple tethered cord syndrome, intraspinal lipoma, congenital dermal sinus, split spinal cord malformation, intraspinal cysts, and other diseases, which can lead to defecation and urination disorders, motor and sensory disorders of the lower limbs, deformity of the lower limbs, and other adverse effects (1–3). The incidence rate of spinal neural tube defects is 0.5–2‰ (4). It is of significant clinical value to use prenatal imaging technology to establish ultra-early diagnosis and prognostic assessment of spinal cord neural tube malformation, reduce the birth rate of severe spinal cord neural tube malformations, and improve the prognosis of children with spinal cord neural tube malformation.

Recently, prenatal ultrasound examination remains the preferred method for routine screening of fetal spinal neural tube malformation (5). It has the following advantages: it is economical and safe and involves dynamic observation. However, the spatial and tissue resolutions of ultrasound examination are relatively low, and the imaging quality is poor when factors such as significantly low amniotic fluid, maternal obesity, fetal malposition, late pregnancy, abdominal scar of pregnant women, and fetal bone occlusion exist (6, 7).

In recent years, magnetic resonance imaging (MRI) has improved the scanning time and signal-to-noise ratio, shortened the scanning time significantly, and reduced the influence of artifacts caused by fetal movement (8–10). Thus, MRI has been widely used in clinical practice. Due to its good soft tissue resolution, fetal MRI is an auxiliary method for prenatal ultrasound screening to assess the presence of fetal central nervous system malformations (11). Fetal MRI detects additional central nervous system malformations at 6.2–14.5% (12). Considering that the incidence rate of spinal neural tube defects is low, it is difficult to establish its prenatal diagnosis. Moreover, current studies on fetal MRI and prenatal diagnosis of spinal neural tube defects are limited, with no effective conclusions drawn (13). With specific regards to neural tube development, MRI can help to identify normal patterns, such as persistence of the V ventricle in the conus or abnormal findings that correlate with well-defined genetic syndromes (14, 15). In fact, genetic confirmation of brain malformations can prove to be extremely time-consuming and expensive; therefore, clinicians and radiologists are constantly trying to define precise fetal and neonatal imaging patterns that could potentially correlate specific phenotypes with their genetic mutations (16).

This study is the first to compare the diagnostic coincidence rate of fetal MRI and ultrasound in the prenatal diagnosis of spinal neural tube defects by analyzing a large sample size and to understand the advantages of fetal MRI in the prenatal diagnosis of spinal neural tube defects.

## Methods

### Patient data

A total of 56 fetuses suspected with spinal neural tube defects by prenatal ultrasound were prospectively followed up in the neurosurgery outpatient department of the First Medical Center of People's Liberation Army General Hospital from August 2018 to January 2021. Further fetal MRI examination was performed within 72 h after ultrasound examination. During follow-up, 48 and eight pregnant women continued and terminated their pregnancy, respectively. Forty-two fetuses were born, including 40 and two single and twin pregnancies, respectively. Forty fetuses (16 boys, 24 girls) who were born in single pregnancies were selected as the study subjects. The age of pregnant women ranged from 21 to 42 (average, 28) years. Gestational age ranged from 20^+2^ to 37^+5^ (average, 28^+2^) weeks. This study conformed to the requirements of the Declaration of Helsinki. Before the study, the family members had fully communicated with each other about the content and significance of this study and signed the informed consent forms. This study was approved by the hospital's ethics committee.

### Prenatal ultrasonography

LOGIQ 9 and Voluson E8 ultrasonic devices manufactured by GE of the USA were used for scanning at 3.5–5.0 Hz (6, 11). By observing the lamina closure of the fetus, position and shape of the conus spinal cord, shape of the spinal canal, and relationship between the end of the conus spinal cord and spinal canal, the fetal spinal neural tube malformation was determined.

### Fetal magnetic resonance imaging

Siemens Spectra 3.0T MRI machine, 6-channel phased array surface coil, breath-hold scan (layer thickness, 3–5 mm; layer spacing, 0–30 mm; field of view, 380 × 380 mm; excitation, 1), half-Fourier acquisition single-shot turbo-spin-echo (HASTE), and true fast imaging with steady-state precession (true FISP) sequence were used for T2-weighted imaging. T1-weighted three-dimensional disturbing phase gradient echo sequencing, Siemens' volume interpolated breath-hold examination (VIBE), and coronal, sagittal, and axial scanning were performed. HASTE had the following parameters: repetition time (TR), 1,200 ms; echo time (TE), 98 ms; flip angle (FA), 120; and matrix, 256 × 256, with the pregnant women holding their breath for 17–20 s at a time. True FISP had the following parameters: TR, 486.77 ms; TE, 1.54 ms; FA, 52; and matrix, 256 × 256, with the pregnant women holding their breath 20 s at a time. VIBE had the following parameters: TR, 3.86 ms; TE, 1.36 ms; and FA, 9.0, with the pregnant women holding their breath 16 s at a time (3, 7, 11). By observing the position and shape of the fetal conus medullaris, the size and shape of the intraspinal mass, its relationship with the conus, and the movement of the spinal cord and nerves, the spinal neural tube malformation in the fetus was determined.

### Postnatal MRI

Siemens Spectra 3.0T MRI was used. Since the lesions in this group were all located at the lumbosacral level, lumbosacral MRI scan was performed within 1 month after birth.

### Analytical method

Forty single pregnancy fetuses were selected for study and analysis. MRI of the lumbosacral vertebrae was performed within 1 month after birth. The coincidence rates of prenatal diagnosis between ultrasound and fetal MRI were compared using postnatal MRI as the standard. This work was jointly completed by a senior and experienced physician from pediatric neurosurgery, imaging and ultrasound departments.

### Statistical method

Data were statistically analyzed using the Statistical Package for the Social Sciences version 26.0. The data in this study were typical independent 2 × 2 contingency tables, the outcome variables were qualitative dichotomies, and the sample size was <40, which met the conditions for the use of Fisher's exact probability test. Thus, Fisher's exact probability test was performed on the four-grid table to analyze the difference in the diagnostic coincidence rate of ultrasonography and fetal nuclear magnetic resonance for different types of spinal cord neural tube malformations. The difference was statistically significant at *P* < 0.05.

## Results

MRI revealed spina bifida, intraspinal lipoma, simple spinal tethered cord syndrome, congenital dermal sinus, skin mass, intraspinal cyst, split spinal cord malformation, lipoma myelomeningocele, and sacrococcygeal teratoma in 28, 19, 10, 8, 8, 4, 3, 2, and 2 fetuses, respectively (Table 1).

**Table 1:** The number and proportion of different types of spinal cord neural tube defects diagnosed by magnetic resonance imaging after birth.

**Disease type**	**Number (case)**	**Proportion (%)**
Spina bifida	28	70%
Intraspinal lipoma	19	47.5%
Simple spinal tethered cord syndrome	10	25%
Congenital dermal sinus	8	20%
Skin mass	8	20%
Intraspinal cyst	4	10%
Split cord malformation	3	7.5%
Lipoma myelomeningocele	2	5%
Sacrococcygeal teratoma	2	5%

*A fetus may have multiple spinal tube defects*.

**Figure 1:**
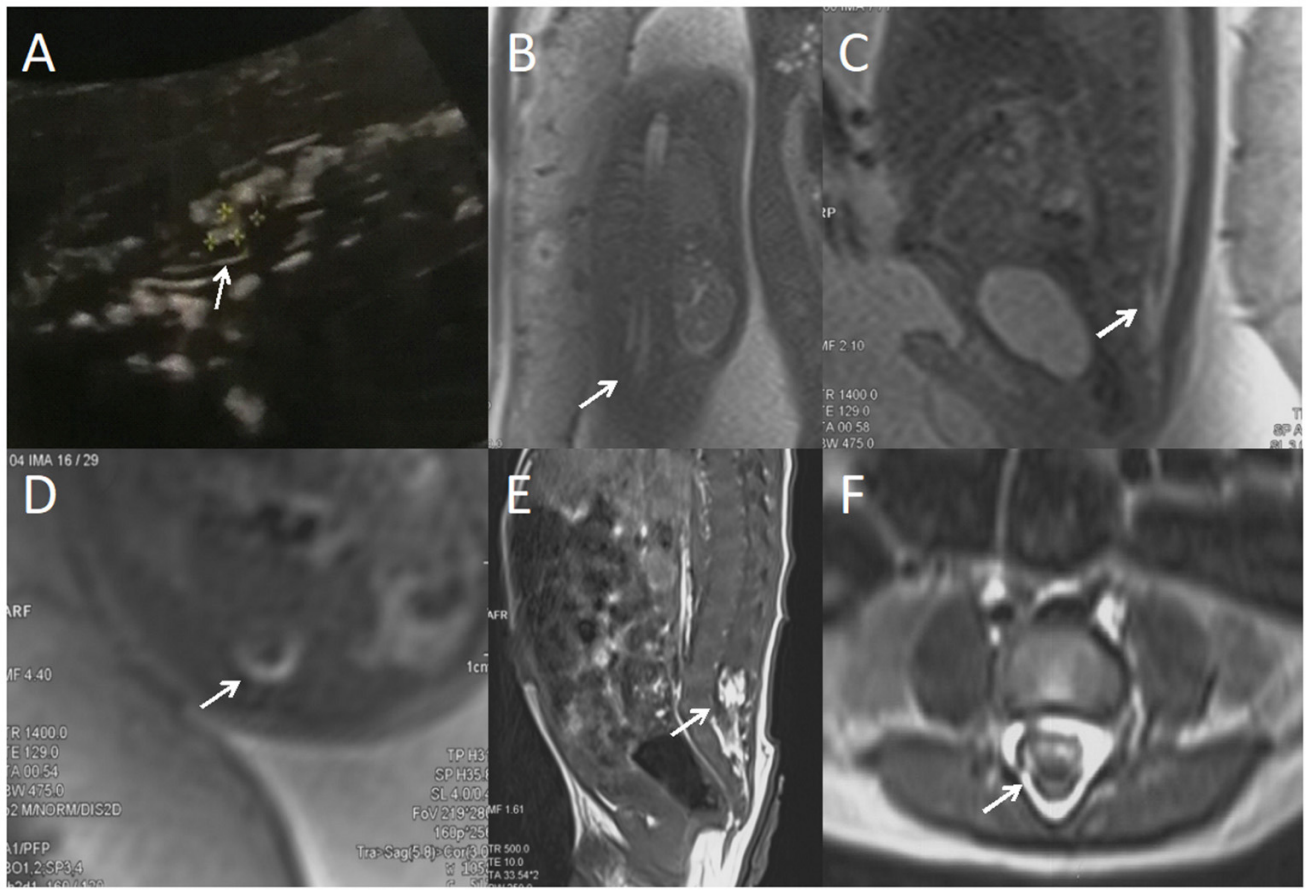
A fetus with intraspinal lipoma. **(A)** the prenatal ultrasound examination at 24 weeks of gestation showed the presence of low conus medullosus in the fetus, hyperechoic mass shadow in the spinal canal, and broken spinal continuity. Thus, spina bifida and intraspinal lipoma were considered. **(B)** Fetal magnetic resonance imaging (MRI) examination at 24 + 2 weeks of gestation showed a coronal view of the low conus medullosus of the fetus, with enlarged end of the conus medullosus and tight adhesion to the posterior edge of the spinal canal. **(C)** Sagittal view showed the low conus of fetal myeloma cord. **(D)** In the axial position, normal subarachnoid space in the spinal canal disappeared, with lipoma and spinal nerves mixed. **(E)** T1 sagittal MRI of the lumbosacral vertebrae 10 days after birth suggested intraspinal lipoma. **(F)** Axial MRI of the lumbosacral vertebrae showed uneven internal signal of lipoma, which was considered to be a hybrid lipoma with the spinal nerves.

**Figure 2:**
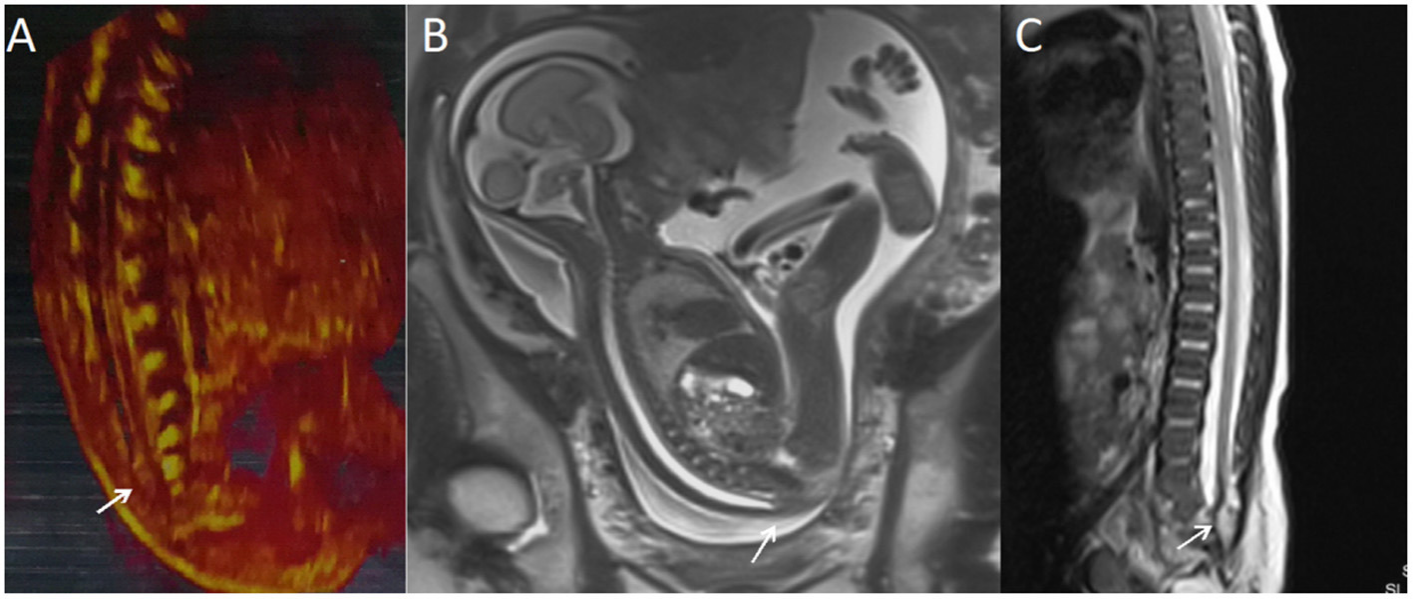
A fetus with intraspinal lipoma. **(A)** The prenatal ultrasound examination at 25 + 1 weeks of gestation showed the presence of low conus medullosus in the fetus and hyperechoic mass shadow in the spinal canal. Thus, intraspinal lipoma was considered. **(B)** Fetal magnetic resonance imaging (MRI) examination at 25 + 2 weeks of gestation showed the low conus medullosus of the fetus, with enlarged end of the conus medullosus and conus terminal and dural sac caudal adhesion. **(C)** T2 sagittal MRI of the lumbosacral vertebrae 20 days after birth suggested spina bifida and intraspinal lipoma.

**Figure 3:**
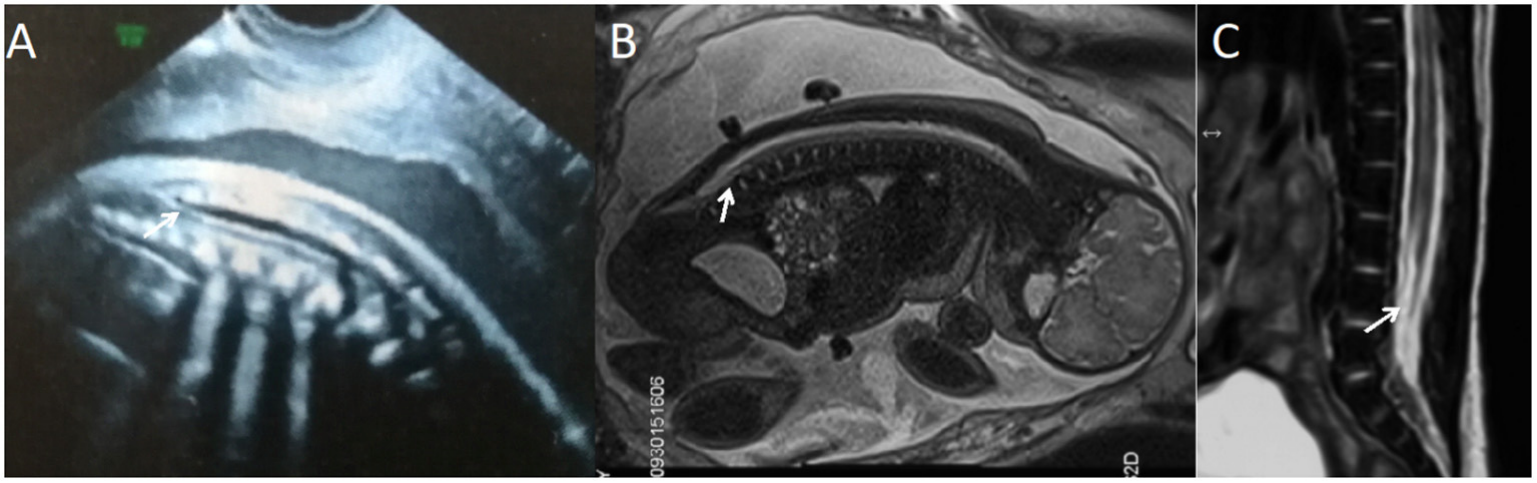
A fetus with simple spinal tethered cord syndrome. **(A)** The prenatal ultrasound examination at 20 + 5 weeks of gestation showed the lower conus medullaris. **(B)** Fetal magnetic resonance imaging (MRI) examination at 20 + 6 weeks of gestation showed the lower conus medullaris. **(C)** T2 sagittal MRI of the lumbosacral vertebrae 22 days after birth suggested simple spinal tethered cord syndrome.

The coincidence rate of prenatal ultrasound for spina bifida was 71.4% (20/28), which was significantly higher than that of fetal MRI (39.2%, 11/28), and the difference was statistically significant (χ^2^ = 7.25, *P* = 0.01 < 0.05). For intraspinal lipoma, the diagnostic coincidence rate of fetal MRI was 73.7% (14/19), which was significantly higher than that of prenatal ultrasound (52.6%) (10/19), and the difference was statistically significant (χ^2^ = 7.54, *P* = 0.01 < 0.05) (Figures 1, 2). For simple spinal tethered cord syndrome, the diagnostic coincidence rate of fetal MRI was 80% (8/10), which was higher than that of prenatal ultrasound (50%) (5/10), but the difference was not statistically significant (χ^2^ = 2.50, *P* = 0.44 > 0.05) (Figure 3). The diagnostic coincidence rate of prenatal ultrasound for congenital dermal sinus was 62.5% (5/8), which was higher than that of fetal MRI (25%) (2/8), and the difference was not statistically significant (χ^2^ = 1.60, *P* = 0.46 > 0.05).The diagnostic coincidence rate of prenatal ultrasound for fetal skin mass was 75% (6/8), which was higher than that of fetal MRI (25%) (2/8), and the difference was not statistically significant (χ^2^ = 0.89, *P* = 1.00 > 0.05). The diagnostic coincidence rate of prenatal ultrasound for intraspinal cyst was 75% (3/4), which was higher than that of fetal MRI (25%), and there was no significant difference between them (χ^2^ = 0.44, *P* = 1.00 > 0.05). The coincidence rate of prenatal ultrasound in the diagnosis of split spinal cord malformation was 66.7% (2/3), which was higher than that of fetal MRI (33.3%), and the difference was not statistically significant (χ^2^ = 0.75, *P* = 1.00 > 0.05). Due to the limited sample size, statistical analyses of lipoma myelomeningocele and sacrococcygeal teratoma were not possible (Table 2).

**Table 2:** Comparative analysis data of the diagnostic coincidence rates of prenatal ultrasound (US) and fetal magnetic resonance imaging (MRI) for different types of spinal neural tube defects.

**Disease type**	**Neonatal MRI**	**Prenatal US**	**Fetal MRI**	**χ^2^**	***P***
		**Diagnose accordance rate (** ***n*** **, %)**			
Spina bifida	28	20 (71.4%)	11 (39.2%)	7.25	0.01
Intraspinal lipoma	19	10 (52.6%)	14 (73.7%)	7.54	0.01
Simple spinal tethered cord syndrome	10	5 (50%)	8 (80%)	2.50	0.44
Congenital dermal sinus	8	5 (62.5%)	2 (25%)	1.60	0.46
Skin mass	8	6 (75%)	2 (25%)	0.89	1.00
Intraspinal cyst	4	3 (75%)	1 (25%)	0.44	1.00
Split cord malformation	3	2 (66.7%)	1 (33.3%)	0.75	1.00
Lipoma myelomeningocele	2	1 (50%)	2 (100%)	No	No
Sacrococcygeal teratoma	2	1 (50%)	2 (100%)	No	No

*Using neonatal MRI as the standard, the difference of the diagnostic coincidence rate between prenatal ultrasound and fetal MRI was statistically analyzed using Fisher's exact probability test and the χ^2^ test, with P < 0.05 suggesting a statistical difference*.

## Discussion

Early and accurate prenatal diagnosis of spinal neural tube defects is significantly important to improve the prenatal diagnostic rate of fetuses with spinal neural tube malformation, help clinicians to formulate a reasonable surgical opportunity for continuing pregnancy of fetuses, and provide preoperative guidance for intrauterine surgical treatment of neural tube dysfunction (2, 3, 6, 10).

Recently, prenatal ultrasound examination remains the preferred screening method to assess the presence of fetal spinal cord neural tube malformation (5). Simultaneously, due to its relatively low spatial and tissue resolutions and the presence of oligohydramnios, maternal obesity, fetal malposition, late pregnancy, abdominal scar in pregnant women, and fetal bone occlusion, its imaging quality development is poor (3, 6, 7). Thus, several prenatal fetal spinal cord neural tube defects are not detected. Fetal MRI study began in 1983 (17). Initially, due to technical limitations, MRI takes a long period of time to collect images, making it difficult to collect clear fetal images (8, 9). In recent years, MRI has improved its scanning time and signal-to-noise ratio, shortened the scanning time significantly, reduced the influence of fetal artifact caused by fetal movement, and greatly improved the clarity of fetal image (8–10). We concluded that fetal MRI can provide excellent soft tissue and spatial resolutions (11), and multidirectional scanning in sagittal, coronal, and axial positions can clearly display the relationship between tissues, with a large field of vision, and can display the overall photo of the fetus (18–22).

Our results suggest that fetal MRI is superior to prenatal ultrasound in the prenatal diagnosis of intraspinal lipoma. The reason is that ultrasound has high sensitivity to water-based and cystic structures and low sensitivity to solid masses in the spinal canal. The occlusion of the fetal lamina and other bones has a certain influence on the acquisition of spinal canal information by ultrasound (2). Fetal MRI has higher spatial resolution and can be scanned in multiple directions, which can develop the size, shape, and location of solid masses in the spinal canal and their relationship with the spinal nerves more clearly (23). In this study, five of the 19 fetuses with intraspinal lipoma were not successfully diagnosed by fetal MRI. The reason lies in the small size of lipoma. The lipoma is located at the distal conus of the spinal cord and reaches the level of the caudal dural sac, where it is attached to subcutaneous fat. The lipoma was located on the dorsal side of the spinal cord and did not adhere to the posterior edge of the spinal canal. This is similar to the study results of Thorne et al. (24). Regarding the classification and diagnosis of intraspinal lipoma, it is difficult to clearly distinguish the specific classification of intraspinal lipoma through current prenatal ultrasound and fetal MRI technology. This will be our area of focus in our future study. However, the findings of this study suggest that ultrasound has its unique advantages in the prenatal diagnosis of spina bifida. This result is consistent with the study results of Wang et al. (13) and Blaicher et al. (25). The reason is that MRI of the bone is unclear, and ultrasound imaging of the fetal spinal ossification center is clearer (26, 27). Thus, we hypothesized that prenatal ultrasound in type I longitudinal crack on spinal deformity has more advantages than fetal MRI, which are reflected in the study of Korostyshevskaya et al. (28), but as a result of the longitudinal crack on the incidence of spinal cord malformation, the number of fetuses is less, with errors in the study results. This allows us to continue to collect late cases for verification. The results of this study show that there is no difference between ultrasound and fetal MRI in the prenatal diagnosis of simple spinal tethered cord syndrome, congenital dermal sinus, skin mass, intraspinal cyst, and split spinal cord malformation. We believe that the main reason for this result is the insufficient sample size. We performed subjective analysis for these types of spinal cord neural tube malformations. In the prenatal diagnosis of simple spinal tethered cord syndrome, fetal MRI can more intuitively display the shape and position of the conus spinal cord, and it is easier to determine the level of the conus spinal cord. In the prenatal diagnosis of congenital dermal sinus, especially for congenital dermal sinus with small fistulas, it is easy to be ignored in prenatal ultrasound and fetal MRI examination, but ultrasound has its unique advantages in the prenatal diagnosis of congenital dermal sinus. Since the fetus can be observed from different angles according to the change of the pregnant woman's position during ultrasound scanning, the skin integrity of the lower back can be observed in the coronal position of the fetus. If the skin continuity is interrupted, the skin has strong echoes outside the skin, and there is a cord-shaped fistula with strong echoes running into the spinal canal. The existence of congenital dermal sinus should be highly suspected. Fetal MRI is not sensitive to the prenatal diagnosis of congenital dermal sinus, unless the fistula of the congenital dermal sinus is large or the growth of exophytes is observed. The fistula shadow of T_2_ phase low signal and the communication between the spinal canal and the outside of the skin can be observed, and there are dermatophyte changes on the outside of the skin. This was the case in the two fetuses correctly diagnosed before fetal MRI in this group. In the diagnosis of skin mass by prenatal ultrasound, the best angle to observe the skin on the back of the fetus can be found by asking the pregnant woman to change her position, and local uplift of the skin on the back of the fetus can be found (10, 24). Good skin integrity and consistent echo of subcutaneous tissue can be distinguished from meningocele and myelomeningocele.

**Figure 4:**
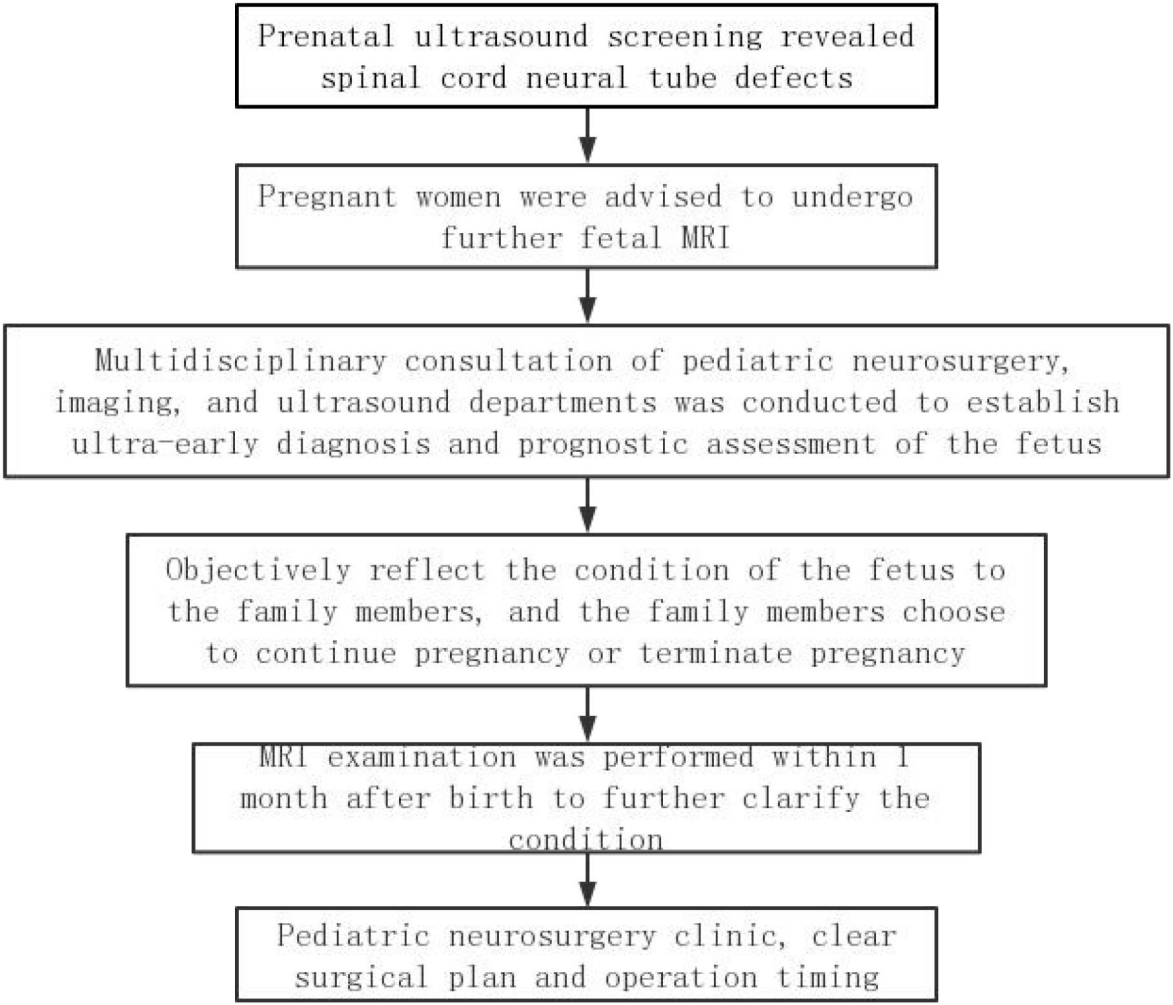
The diagnosis and treatment procedures for spinal cord neural tube defects.

Recently, there are no standardized diagnosis and treatment plan for the fetus with spinal neural tube defects worldwide. A complete set of diagnosis and treatment procedures can improve the prenatal diagnostic rate of spinal cord neural tube defects, reduce the abortion rate of mild spinal cord neural tube malformations, reduce the birth rate of severe spinal cord neural tube malformations, establish a reasonable operation time for the fetus to continue pregnancy, and provide preoperative guidance for the intrauterine surgical treatment of neural tube insufficiency. Therefore, we summarized a set of diagnosis and treatment procedures for reference based on our study experience (Figure 4).

## Limitations

This study has some limitations. First, the prenatal diagnosis of some types of spinal cord neural tube malformations is not convincing due to the limited sample size of this study considering the few and precious cases of spinal cord neural tube malformations found in prenatal examination. Secondly, fetal MRI examination is not the preferred examination for prenatal screening. In this study, fetal MRI was a supplementary examination after prenatal ultrasound suspected the presence of spinal cord neural tube malformation in the fetus, and there was a certain error in the diagnostic coincidence rate.

## Conclusion

Fetal MRI can be used as an important supplement to ultrasound in the prenatal diagnosis of spinal cord neural tube defects. Fetal MRI has an advantage over ultrasound in detecting intraspinal lipoma. Ultrasound has an advantage over fetal MRI in detecting spina bifida. The discussion and cooperation of neurosurgery, ultrasound, and imaging departments are significantly important for the early diagnosis of fetal spinal cord neural tube defects.

## Data availability statement

The raw data supporting the conclusions of this article will be made available by the authors, without undue reservation.

## Ethics statement

The studies involving human participants were reviewed and approved by Chinese PLA General Hospital. Written informed consent to participate in this study was provided by the participants' legal guardian/next of kin.

## Author contributions

GG: conceptualization, methodology, and writing—original draft preparation. BT and YC: formal analysis, validation, and visualization. JY, MS, HW, and FH: software, data curation, and investigation. SL and MW: data curation and supervision. AS: writing—review and editing and resources. All authors contributed to the article and approved the submitted version.

## Funding

This work was supported by the National Key Research and Development Program (grant number SQ2018YFC100110) and the Capital's Funds for Health Improvement and Research (grant number CFH2022-2-5022).

## Conflict of interest

The authors declare that the research was conducted in the absence of any commercial or financial relationships that could be construed as a potential conflict of interest.

## Publisher's note

All claims expressed in this article are solely those of the authors and do not necessarily represent those of their affiliated organizations, or those of the publisher, the editors and the reviewers. Any product that may be evaluated in this article, or claim that may be made by its manufacturer, is not guaranteed or endorsed by the publisher.
